# *SF3B1* mutations induce R-loop accumulation and DNA damage in MDS and leukemia cells with therapeutic implications

**DOI:** 10.1038/s41375-020-0753-9

**Published:** 2020-02-19

**Authors:** Shalini Singh, Doaa Ahmed, Hamid Dolatshad, Dharamveer Tatwavedi, Ulrike Schulze, Andrea Sanchi, Sarah Ryley, Ashish Dhir, Lee Carpenter, Suzanne M. Watt, David J. Roberts, Amal M. Abdel-Aal, Sohair K. Sayed, Somaia A. Mohamed, Anna Schuh, Paresh Vyas, Sally Killick, Andriana G. Kotini, Eirini P. Papapetrou, Daniel H. Wiseman, Andrea Pellagatti, Jacqueline Boultwood

**Affiliations:** 1 Bloodwise Molecular Haematology Unit, Nuffield Division of Clinical Laboratory Sciences, Radcliffe Department of Medicine, University of Oxford, Oxford, UK; 2 Clinical Pathology Department, Assiut University, Assiut, Egypt; 3 Weatherall Institute of Molecular Medicine, Radcliffe Department of Medicine, University of Oxford, Oxford, UK; 4 Oxford Medical Genetics Laboratories, Oxford University Hospitals NHS Foundation Trust, The Churchill Hospital, Oxford, UK; 5 Centre for Genomic and Experimental Medicine, MRC Institute of Genetics and Molecular Medicine, University of Edinburgh, Edinburgh, UK; 6 Stem Cell Research, Nuffield Division of Clinical Laboratory Sciences, Radcliffe Department of Medicine, University of Oxford, Oxford, UK; 7 National Health Service Blood and Transplant, John Radcliffe Hospital, Oxford, UK; 8 Molecular Diagnostic Centre, Department of Oncology, University of Oxford, Oxford, UK; 9 MRC Molecular Hematology Unit, WIMM, University of Oxford, Oxford, UK; 10 Haematology Theme Oxford Biomedical Research Centre and Department of Hematology, Oxford University Hospitals NHS Foundation Trust, Oxford, UK; 11 Department of Haematology, Royal Bournemouth Hospital, Bournemouth, UK; 12 Department of Oncological Sciences, Icahn School of Medicine at Mount Sinai, New York, NY USA; 13 Tisch Cancer Institute, Icahn School of Medicine at Mount Sinai, New York, NY USA; 14 Black Family Stem Cell Institute, Icahn School of Medicine at Mount Sinai, New York, NY USA; 15 Department of Medicine, Icahn School of Medicine at Mount Sinai, New York, NY USA; 16 Division of Cancer Sciences, University of Manchester, Manchester, UK

**Keywords:** Myelodysplastic syndrome, Leukaemia

## To the Editor:

The myelodysplastic syndromes (MDS) are common myeloid malignancies characterized by ineffective hematopoiesis and blood cytopenias, with patients showing increasing bone marrow blasts with disease progression [1]. Mutations in genes involved in pre-mRNA splicing (*SF3B1, SRSF2, U2AF1,* and *ZRSR2*) are the most common mutations found in MDS, occurring in over 50% of all cases [2–4]. There is evidence that some spliceosome components play a role in the maintenance of genomic stability [5]. Splicing is a transcription coupled process; splicing factor mutations affect transcription and may lead to the accumulation of R-loops (RNA-DNA hybrids with a displaced single stranded DNA) [6]. Mutations in the splicing factors *SRSF2* and *U2AF1* have been recently shown to increase R-loop formation in leukemia cell lines, resulting in increased DNA damage, replication stress, and activation of the ATR-Chk1 pathway [7, 8]. *SF3B1* is the most frequently mutated splicing factor gene in MDS, with mutations occurring in 25–30% of MDS patients [9–11]. *SF3B1* mutations are also found at lower frequency in other hematological malignancies [4, 11] and in some individuals with clonal hematopoiesis of indeterminate potential [12]. A role of *SF3B1* mutations in R-loop accumulation and DNA damage has not yet been reported in hematopoietic cells. Here, we investigated the effects of *SF3B1* mutations on R-loop formation and associated DNA damage response in MDS and leukemia cells, and we also explored potential therapeutic implications.

**Fig. 1:**
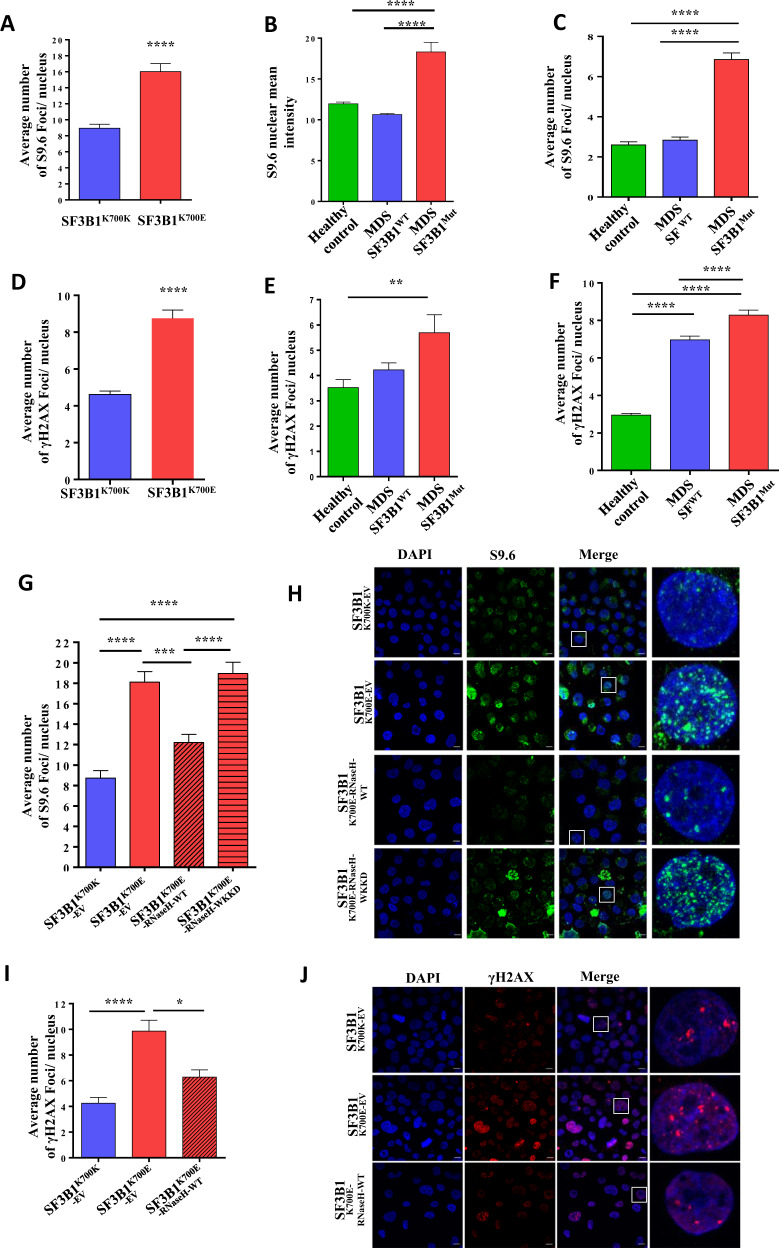
*SF3B1* mutation leads to increased R-loops and associated DNA damage response. Quantitative analysis of S9.6 foci detected by immunocytochemistry in (**a**) SF3B1^K700K^ and SF3B1^K700E^ K562 cells (*n* > 300 cells/category) (**b**) iPSC clones generated from an MDS patient sample with *SF3B1* mutation (MDS SF3B1^Mut^: iPSC clone harboring the *SF3B1* mutation; MDS SF3B1^WT^: iPSC clone without the *SF3B1* mutation) and from a healthy control (*n* > 150 cells/category) (**c**) CD34+ cells from MDS patients with *SF3B1* mutation (MDS SF3B1^Mut^), from MDS patients without splicing factor mutations (MDS SF^WT^), and from healthy controls (*n* > 300 cells/category). Quantitative analysis of γ-H2AX foci detected by immunocytochemistry in (**d**) SF3B1^K700K^ and SF3B1^K700E^ K562 cells (*n* > 300 cells/category) (**e**) iPSC clones generated from an MDS patient sample with *SF3B1* mutation (MDS SF3B1^Mut^: iPSC clone harboring the *SF3B1* mutation; MDS SF3B1^WT^: iPSC clone without the *SF3B1* mutation) and from a healthy control (*n* > 100 cells/category) (**f**) CD34+ cells from MDS patients with *SF3B1* mutation (MDS SF3B1^Mut^), from MDS patients without splicing factor mutations (MDS SF^WT^), and from healthy controls (*n* > 150 cells/category). **g** Quantitative data and **h** representative images of S9.6 foci detected by immunocytochemistry in SF3B1^K700K-EV^, SF3B1^K700E-EV^, SF3B1^K700E-RNaseH-WT^, and SF3B1^K700E-RNaseH-WKKD^ K562 cells (*n* > 300 cells/category). Green- S9.6; blue- DAPI. **i** Quantitative analysis and **j** representative images of γ-H2AX foci in SF3B1^K700K-EV^, SF3B1^K700E-EV^, and SF3B1^K700E-RNaseH-WT^ K562 cells (*n* > 150 cells/category). Red- γ-H2AX; blue: DAPI. All values are plotted as mean ± SEM. *P* values in **a** and **d** were obtained using the unpaired Student’s *T* test. *P* values in **b**–**c**, **e**–**g**, **i** were obtained using one-way ANOVA with the Tukey’s multiple comparisons test. *P < 0.05; **P < 0.01; ***P < 0.001; ****P < 0.0001. Scale bar −10 µm.

Firstly, we investigated the effects of *SF3B1* mutations on the formation of R-loops, as measured by immunofluorescence staining using the S9.6 antibody (Supplementary Materials and Methods) [7]. K562 cells (a myeloid leukemia cell line) with the SF3B1^K700E^ mutation showed a significant increase in the number of S9.6 foci, indicating increased R-loops, compared with isogenic SF3B1^K700K^ K562 cells (Fig. 1a, S1A). We then analyzed induced pluripotent stem cells (iPSCs) that we generated (and characterized, Fig. S2) from bone marrow CD34+ cells of one *SF3B1* mutant MDS patient and of one healthy control (Supplementary Materials and Methods). A significant increase in R-loops was observed in an iPSC clone harboring the *SF3B1* mutation compared with another iPSC clone without the *SF3B1* mutation obtained from same MDS patient, and to iPSCs from the healthy control (Fig. 1b). We have also analyzed the levels of R-loops in the bone marrow CD34+ cells from three *SF3B1* mutant MDS patients, three splicing factor wildtype MDS patients, and three healthy controls. Importantly, CD34+ cells from *SF3B1* mutant MDS patients (Table S1) showed a significant and marked increase in R-loops compared with CD34+ cells from MDS patients without splicing factor mutations (2.4-fold) and from healthy controls (2.6-fold) (Fig. 1c, S1B). Our results demonstrate that an accumulation of R-loops occurs in association with the presence of *SF3B1* mutations in MDS and leukemia cells.

We then investigated the effects of *SF3B1* mutations on the DNA damage response, as measured by immunofluorescence staining using anti-γ-H2AX antibody (Supplementary Materials and Methods) [7]. K562 cells with the SF3B1^K700E^ mutation showed a significant increase in the number of γ-H2AX foci, indicating increased DNA damage, compared with isogenic control SF3B1^K700K^ K562 cells (Fig. 1d, S3A). Similarly, an iPSC clone harboring the *SF3B1* mutation showed increased DNA damage as compared with another iPSC clone without the *SF3B1* mutation obtained from same MDS patient and to iPSCs from a healthy control (Fig. 1e). Bone marrow CD34+ cells from *SF3B1* mutant MDS patients showed significantly increased DNA damage compared with CD34+ cells from MDS patients without splicing factor mutations and from healthy controls (Fig. 1f, S3B).

To investigate whether the observed DNA damage in *SF3B1* mutant K562 cells results from induced R-loops, we overexpressed RNaseH1 (encoding an enzyme that degrades the RNA in RNA:DNA hybrids) to resolve R-loops in these cells. RNaseH1 overexpression significantly reduced the number of S9.6 foci in SF3B1^K700E^ K562 cells compared with SF3B1^K700E^ K562 cells expressing an empty vector (Fig. 1g, h). Furthermore, SF3B1^K700E^ K562 cells expressing WKKD mutant RNaseH1 (lacking hybrid binding and RNaseH1 activity) did not show a decrease in the number of S9.6 foci (Fig. 1g, h). RNaseH1 overexpression also significantly reduced the number of γ-H2AX foci (Fig. 1i, j) in SF3B1^K700E^ K562 cells compared with SF3B1^K700E^ K562 cells expressing an empty vector. Further, western blot analysis of γ-H2AX levels in SF3B1^K700E^ K562 cells expressing RNaseH1 also showed decreased levels of γ-H2AX (Fig. S4). These data demonstrate that increased levels of R-loops result in increased DNA damage in *SF3B1* mutant leukemia cells.

Next, to investigate the signaling events related to DNA damage in *SF3B1* mutant cells, we have studied ATR and ATM signaling, two pathways that are frequently activated following DNA damage [13]. The ATR signaling pathway was analyzed by measuring the levels of Chk1 phosphorylation at Ser345, a hallmark of activation of the ATR pathway, in K562 cells (Supplementary Materials and Methods). We observed increased phosphorylation of Chk1 in K562 cells with the SF3B1^K700E^ mutation compared with isogenic SF3B1^K700K^ K562 cells (Fig. S5A). Suppression of R-loops by RNaseH1 overexpression resulted in decreased Chk1 phosphorylation, indicating suppression of ATR pathway activation in SF3B1^K700E^ K562 cells (Fig. S5A). In contrast, we did not observe activation of the ATM signaling pathway in the SF3B1^K700E^ K562 cells as analyzed by measuring the levels of ATM phosphorylation at Ser1981, Chk2 phosphorylation at Thr68, and RPA32 phosphorylation at Ser4/8 (Fig. S5B). These data demonstrate that *SF3B1* mutations are associated with downstream activation of ATR but not ATM signaling.

**Fig. 2:**
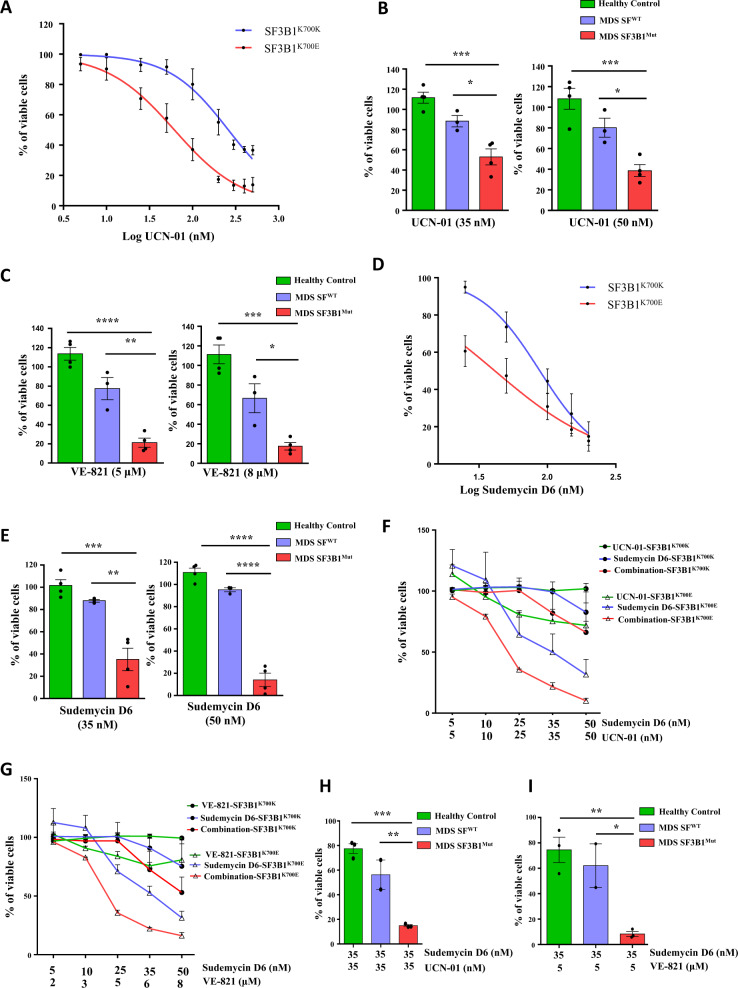
SF3B1 mutant cells are preferentially sensitive to UCN-01, VE-821, and Sudemycin D6 and the effects of UCN-01 and VE-821 were synergistically enhanced by Sudemycin D6. **a** Viability of SF3B1^K700K^ and SF3B1^K700E^ K562 cells treated with UCN-01. IC_50_ obtained for SF3B1^K700K^ and SF3B1^K700E^ K562 cells are 260.8 ± 1.05 nM and 61.53 ± 1.1 nM, respectively (*n* = 3). Effect of (**b**) UCN-01 and (**c**) VE-821 treatment on viability of CD34+ cells from MDS SF3B1^Mut^ (*n* = 4), from MDS SF^WT^ (*n* = 3), and from healthy controls (*n* = 4). **d** Viability of SF3B1^K700K^ and SF3B1^K700E^ K562 cells treated with the splicing modulator Sudemycin D6. IC_50_ obtained for SF3B1^K700K^ and SF3B1^K700E^ K562 cells are 87.8 ± 1.09 nM and 41.1 ± 1.15 nM, respectively (*n* = 3). **e** Effect of Sudemycin D6 on viability of CD34+ cells from MDS SF3B1^Mut^ (*n* = 4), from MDS SF^WT^ (*n* = 3), and from healthy control (*n* = 4). Effect of combination of different doses of (**f**) Sudemycin D6 and UCN-01 (*n* = 2) and (**g**) Sudemycin D6 and VE-821 (*n* = 2) on viability of SF3B1^K700K^ and SF3B1^K700E^ K562 cells. Effect of combination of (**h**) Sudemycin D6 (35 nM) and UCN-01 (35 nM) and (**i**) Sudemycin D6 (35 nM) and VE-821 (5 µM) on viability of CD34+ cells from MDS SF3B1^Mut^ (*n* = 3), from MDS SF^WT^ (*n* = 2), and from healthy controls (*n* = 3). All values are plotted as mean ± SEM. Results shown in **a** and **d** were analyzed by using nonlinear regression. *P* values for **b**–**c**, **e**, **h**–**i** are calculated using one-way ANOVA with the Tukey’s multiple comparisons test. *P < 0.05; **P < 0.01; ***P < 0.001; ****P < 0.0001.

We sought to explore the functional importance of ATR activation associated with *SF3B1* mutation and determine whether this could represent a therapeutic vulnerability. We evaluated the sensitivity of *SF3B1* mutant cells to VE-821 (Supplementary Materials and Methods). SF3B1^K700E^ K562 cells showed preferential sensitivity to the ATR inhibitor VE-821 compared with isogenic SF3B1^K700K^ K562 cells (Fig. S6A). Chk1 is a critical substrate of ATR, and we next chose to investigate the effects of Chk1 inhibition in SF3B1^K700E^ K562 cells. Interestingly, SF3B1^K700E^ K562 cells demonstrated preferential sensitivity to the Chk1 inhibitor UCN-01 compared with SF3B1^K700K^ K562 cells, suggesting that ATR-Chk1 activation is important for the survival of *SF3B1* mutant cells (Fig. 2a). The effect of RNaseH1 overexpression on the sensitivity of SF3B1^K700E^ K562 cells towards UCN-01 was also examined. We found that the sensitivity of SF3B1^K700E^ K562 towards UCN-01 decreased after overexpressing RNaseH1 (Fig. S6B). Treatment with an ATM inhibitor (KU-55933) did not show a significant difference in the sensitivity of SF3B1^K700E^ K562 cells compared with isogenic SF3B1^K700K^ K562 cells (Fig. S6C). Notably, bone marrow CD34+ cells from SF3B1 mutant MDS patients showed preferential sensitivity towards UCN-01 (Fig. 2b) and VE-821 (Fig. 2c) compared with CD34+ cells from MDS patients without splicing factor mutations and from healthy controls. These results show that activation of ATR, but not ATM, plays an important role for the survival of *SF3B1* mutant cells, and that these cells are vulnerable to Chk1 inhibition.

Preferential sensitivity of splicing factor mutant cells towards splicing modulators has been reported previously [4, 14, 15]. Thus, we investigated whether a splicing modulator could increase the sensitivity of *SF3B1* mutant cells to ATR or Chk1 inhibition. The splicing modulator Sudemycin D6 has been shown to preferentially kill U2AF1 mutant cells [14], but its effects on myeloid leukemia cells with the *SF3B1* mutation have not been evaluated. Here, we showed that SF3B1^K700E^ K562 cells were preferentially sensitive to Sudemycin D6 compared with isogenic SF3B1^K700K^ K562 cells (Fig. 2d). Bone marrow CD34+ cells from *SF3B1* mutant MDS patients showed preferential sensitivity to Sudemycin D6 compared with CD34+ cells from MDS patients without splicing factor mutations and from healthy controls (Fig. 2e). We then tested the effects of Sudemycin D6 in combination with an ATR inhibitor or a Chk1 inhibitor. The effects of VE-821 and UCN-01 on *SF3B1* mutant K562 cells were enhanced by Sudemycin D6 (Fig. 2f, g). We have also determined the synergy scores of Sudemycin D6 and UCN-01 (Fig. S7A, B), and Sudemycin D6 and VE-821 (Fig. S7C, D) on SF3B1^K700K^ and SF3B1^K700E^ K562 cells. Various dose combinations showed a positive synergy score (δ-score), indicating synergy of Sudemycin D6 with VE-821 and UCN-01, with higher scores for SF3B1^K700E^ K562 cells. Importantly, bone marrow CD34+ cells from *SF3B1* mutant MDS patients also showed preferential sensitivity towards the combination of Sudemycin D6 with UCN-01 (Fig. 2h) or with VE-821 (Fig. 2i).

In summary, we show for the first time that mutations of *SF3B1*, the most commonly mutated splicing factor gene in MDS [2, 3, 9, 11], lead to the accumulation of R-loops and associated DNA damage, resulting in activation of the ATR pathway in MDS and leukemia cells. The suppression of R-loops rescued cellular defects including DNA damage and ATR-Chk1 activation. Our current study on mutant *SF3B1*, and previous studies by others on mutant *U2AF1* and *SRSF2* [7, 8], together demonstrate that different mutated splicing factors in hematopoietic cells all have convergent effects on R-loop elevation leading to DNA damage. It is possible that this R-loop induced DNA damage may give rise to deleterious mutations in MDS hematopoietic stem and progenitor cells, contributing to the clonal advantage of splicing factor mutant cells in human bone marrow. Future studies seeking to compare R-loop levels in the CD34+ cells of *SF3B1* mutant MDS cases with those in CD34+ cells of MDS patients with mutations of other splicing factors (*SRSF2, U2AF1, ZRSR2*) are warranted.

This is the first study showing that splicing factor mutant MDS and leukemia cells are preferentially sensitive to the Chk1 inhibitor UCN-01, suggesting that Chk1 inhibition, alone or in combination with splicing modulators, may represent a novel therapeutic strategy to target splicing factor mutant cells. This strategy could also be potentially extended to therapeutically target other types of cancers known to harbor *SF3B1* mutations. This study provides preclinical evidence that MDS patients with spliceosome mutations may benefit from Chk1 inhibition to exploit the R-loop-associated vulnerability induced by these mutations.

## Supplementary information

Supplementary Materials and Methods

Table S1

Supplementary Figure Legends

Figure S1

Figure S2

Figure S3

Figure S4

Figure S5

Figure S6

Figure S7
