# Supplementary material for: SF3B1 mutations induce R-loop accumulation and DNA damage in MDS and leukemia cells with therapeutic implications
Source: Leukemia. 2020 Feb 19;34(9):2525–30. doi: 10.1038/s41375-020-0753-9 (PMC7449882; doi:10.1038/s41375-020-0753-9)
Supplement: Supplementary file 1 — Supplementary Materials and Methods [file 41375_2020_753_MOESM1_ESM.docx]

**Supplementary Materials and Methods**

**Cell lines**

K562 cells with the SF3B1^K700E^ mutation and isogenic SF3B1^K700K^ K562 cells were purchased from Horizon Discovery. K562 cells were cultured in RPMI 1640 medium (Sigma-Aldrich, St. Louis, Missouri, United States) containing 10% fetal bovine serum at 37 °C and 5% CO_2_. ppyCAG_RNaseH1_WT (Addgene plasmid#111906, http://n2t.net/addgene:111906; RRID:Addgene_111906) and ppyCAG_RNaseH1_WKKD (Addgene plasmid#111905, http://n2t.net/addgene:111905; RRID:Addgene_111905) vectors were gifts from Xiang-Dong Fu. WKKD mutant RNaseH1 (W43A, K59A, K60A and D210N in both the catalytic and binding domains) lack hybrid binding and RNaseH1 activity [[1](#_ENREF_1)]. Stable K562 cells overexpressing RNaseH1 were generated by transfecting cells with the ppyCAG-RNaseH1 vectors followed by hygromycin selection (200 μg/ml) [[1](#_ENREF_1),[2](#_ENREF_2)]. All cell lines used in this study tested negative for mycoplasma.

**Patient samples**

Bone marrow samples from MDS patients were obtained with informed consent and collected with approval of research ethics committees (Oxford, REC 17/YH/0322; Oxford MDSBio Study, MREC 06/Q1606/110; Manchester Cancer Research Centre Haematological Malignancy Biobank, approval of the South Manchester Research Ethics Committee). Patient samples from Oxford centres were sequenced using a next-generation sequencing myeloid gene panel, as previously described [[3](#_ENREF_3)]. Patient samples from the Manchester centre were subjected to targeted sequencing for recurrent myeloid mutations using a 40-gene panel (Oncomine Myeloid Research Assay; ThermoFisher Scientific, Waltham, Massachusetts, United States) on the Ion Torrent platform. High confidence single nucleotide and small insertion/deletion variants were identified and annotated using a modified version of the inbuilt Ion Reporter Oncomine Myeloid Research DNA Single Sample analysis workflow. Unfiltered variant lists were manually inspected to exclude documented polymorphisms and likely artefactual calls.

**Isolation of CD34^+^ cells from bone marrow mononuclear cells**

Frozen bone marrow mononuclear cells from healthy donors (Stemcell Technologies, Vancouver, Canada) and from MDS patients were thawed and treated with DNase 1 (100U/ml) (ThermoFisher Scientific) in Dulbecco's PBS (pH 7.0-7.2) (Sigma Aldrich), containing 0.5 mM MgCl_2_, 1 mM CaCl_2_ and 0.5% BSA (Sigma Aldrich) for 45 mins at room temperature. CD34^+^ cells were isolated using the CD34 MicroBead Kit UltraPure (Miltenyi Biotec, Bergisch Gladbach, Germany), according to the manufacturer’s protocol. The purity of the isolated CD34^+^ cells was measured by flow cytometry (using CD34-PE, human, Clone: AC136, Miltenyi Biotec) and was >85% for all samples tested. CD34^+^ cells were cultured in StemSpan Serum-Free Expansion Medium (Stemcell Technologies) containing StemSpan CD34^+^ Expansion Supplement (Stemcell Technologies) for 48 hours before performing experiments.

**Generation and characterization of induced pluripotent stem cells (iPSCs)**

*Generation of iPSCs.* Cryopreserved bone marrow CD34^+^ cells of one healthy control (Lonza, Basel, Switzerland) and one MDS patient harboring an *SF3B1* mutation (c.2098A>G, p.K700E) and a *DNMT3A* mutation (c.2645G>A, p.R882H) were thawed and cultured in StemSpan SFEM media (Stemcell Technologies) supplemented with 100 ng/mL Stem Cell Factor, 10 ng/mL IL-3, 100ng/ml Flt3, 40 μg/mL lipids (Stemcell Technologies), 2 mM L-glutamine, and 1% penicillin-streptomycin solution (Sigma Aldrich). All cytokines were obtained from Miltenyi Biotec unless otherwise stated. The cells were cultured for 3-5 days to induce cell proliferation. CytoTune 2.0 Sendai reprogramming kit (Life Technologies) was used to generate iPSCs, according to the manufacturer’s instructions. After 3-4 weeks, multiple colonies with human embryonic stem cell (hESC)-like morphology were picked and expanded. One iPSC clone harboring the *SF3B1* and the *DNMT3A* mutations and one iPSC clone without these mutations from the MDS patient sample, and one iPSC clone from the healthy control sample, were selected for characterization and experimental work. The *SF3B1* mutation status (Fig. S2A) and *DNMT3A* mutation status was confirmed in patient and control iPSCs using Sanger sequencing. iPSC lines were cultured feeder-free in Matrigel (Corning Incorporated, Corning, New York, United States) pre-coated dishes using mTeSR hESC media (Stem Cell Technologies) culture conditions, according to manufacturer’s guidelines in a hypoxic incubator (5% O_2_).

*Confirmation of clearance of Sendai virus reprogramming factors.* The absence of viral transgenes by CytoTune 2.0 Sendai virus in the iPSC genome was evaluated according to manufacturer’s guidelines (Life Technologies, Carlsbad, California, United States). We confirmed that the transduced genes were not expressed at the mRNA level in the established iPSC clones (Fig. S2B) using real-time quantitative PCR.

*Characterization of iPSCs.* The iPSC clones retained typical characteristics of pluripotent stem cells such as expression of immature ES cell markers (SSEA4, TRA181, TRA160), as examined by immunocytochemistry (Fig. S2C) and flow cytometry (Fig. S2D), and the ability to differentiate into the three germ layers *in vitro* (Fig. S2E). Karyotypic analysis by G-banding of the iPSC clones derived from the MDS patient and the healthy control showed a normal karyotype.

*Immunocytochemistry for expression of immature ES cell markers.* iPSCs were grown in 24-well cell culture plates coated with Matrigel until they reached a confluency of 80%. Cells were fixed in 4% paraformaldehyde (ChemCruz biochemical, Santa Cruz Biotechnology, Dallas, Texas, United States) for 15 minutes at room temperature. Fixed cells were washed in PBS and blocking of nonspecific binding sites was performed in 5% BSA (Sigma-Aldrich) in PBS. Cells were incubated with primary antibodies [TRA-1-81 (Stemgent, Cambridge, Massachusetts, United States, Cat 09-0011, 1:100), TRA-1-60 (Merck Millipore, Burlington, Massachusetts, United States, Cat# MAB4360, 1:200), SSEA4 (Merck Millipore, Cat# MAB4304, 1:200)] at 4ºC overnight. Cells were then incubated with anti-mouse antibody (Invitrogen, Carlsbad, California, United States, Cat# A-21202) for 1 h at room temperature in the dark. For nuclear visualization, cells were incubated with 2 µM DAPI (Invitrogen) for 5 minutes. Fluorescence microscopy was performed with an EVOS FL2 Auto fluorescence microscope (Invitrogen) and processed with ImageJ.

*Flow Cytometry for expression of immature ES cell markers.* Cells were washed with PBS and stained with fixable viability dye eFlour 780 (Invitrogen, Cat# 65-0865-14) according to manufacturer’s instructions, and were subsequently fixed in 2% paraformaldehyde (ChemCruz biochemical). Cells were incubated with antibodies [(TRA-1-81 (Invitrogen Cat# 12-8883-82), TRA-1-60-R antibody (BioLegend, San Diego, California, United States, Cat# 330605), SSEA4 (Invitrogen, Cat # 53-8843-42)] for 30 minutes at 4ºC in the dark and washed with PBS. Flow cytometry was performed on a BD LSRII instrument (Becton Dickinson, Franklin Lakes, New Jersey, United States). Data analysis was performed using FlowJo 10 Software version 10.6.1 (Tree Star).

*Trilineage differentiation of iPSCs analysed by flow cytometry*. *In vitro* trilineage differentiation was carried out using STEMdiff Trilineage Differentiation Kit (Stemcell Technologies), according to manufacturer’s guidelines. Cells were washed with PBS and stained with different markers depending on the differentiation lineage. Mesodermal differentiation potential was assessed by the formation of endothelial cells (CD144^+^) (MACS Miltenyi Biotec, Cat# 130-100-713) and the presence of smooth muscle cells (CD140b^+^) (MACS Miltenyi Biotec, Cat# 130-105-322). Endodermal differentiation was assessed by the presence of definitive endoderm cells CD184^+^ (MACS Miltenyi Biotec, Cat# 130-109-886) and Sox17^+^ (MACS Miltenyi Biotec, Cat# 130-111-147). Ectodermal differentiation potential was assessed by the formation of neuroectoderm cells Pax6^+^ (MACS Miltenyi Biotec, Cat# 130-107-829) and Sox2^+^ (MACS Miltenyi Biotec, Cat# 130-104-993). Cells were stained as per manufacturer’s instruction. Flow cytometry was performed on a BD LSRII device instrument (Becton Dickinson). Data analysis was performed using FlowJo 10 Software version 10.6.1 (Tree Star Inc., Ashland, Oregon, USA).

**S9.6 staining for R-loop analysis**

Staining of K562 and CD34^+^ cells was performed by fixing cells in 4% paraformaldehyde for 20 mins. Fixed cells were then cytospun on Corning BioCoat Poly-L-Lysine coverslips. Staining of iPSCs was performed by fixing cells in 100% methanol for 15 mins at 4ᴼC. iPSCs were then washed twice with PBS and ice cold 70% ethanol was added dropwise and cells were incubated on ice for 30 mins. After fixation, cells (iPSCs, K562 and CD34^+^) were blocked with blocking buffer (5% BSA and 0.3% Triton X-100 in PBS) for 1 hr at room temperature. This was followed by incubation with S9.6 antibody (1:100 dilution, Anti-DNA-RNA Hybrid Antibody, clone S9.6, Cat#MABE1095, Merck) overnight at 4°C. On the next day, cells were washed 5 times with wash buffer (1X PBS, 0.03% Triton X-100) and then incubated with fluorescence-conjugated secondary antibody (Alexa Fluor 488 Donkey anti-Mouse IgG (H+L), 1:500 dilutions, Cat#A-21202, ThermoFisher Scientific) for 1hr at room temperature. Cells were then washed thrice with PBS and mounted with Fluoroshield mounting media containing DAPI (Merck) [[2](#_ENREF_2),[4](#_ENREF_4),[5](#_ENREF_5)]. The images were captured using a LSM-880 confocal microscope (Carl Zeiss AG, Oberkochen, Germany) with Plan-Apochromat 63x/1.40 Oil DIC M27 objective and analysed using Fiji software (ImageJ).

**γH2AX staining for DNA damage analysis**

Cells (iPSCs, K562 & CD34^+^) were fixed with 4% paraformaldehyde for 20 min at room temperature. Cells were further incubated with 0.3% Triton X-100 in PBS for 30 min, at room temperature. Cells were then washed thrice in PBS and incubated with blocking buffer (5% BSA and 0.03% Triton X-100 in PBS) for 1 hr at room temperature followed by incubation with primary antibody for γH2AX (1:200 dilution, Anti-phospho-Histone H2A.X (Ser139) Antibody, clone JBW301, Cat#05-636, Merck Millipore) overnight at 4°C. Cells were then washed thrice with PBS and incubated with the fluorescence-conjugated secondary antibody (Alexa Fluor 594 Goat anti-Mouse IgG (H+L), 1:500 dilutions, ThermoFisher Scientific, Cat#A-11005) for 1 hr at room temperature. Cells were then washed thrice with PBS and mounted with Fluoroshield mounting media containing DAPI (Sigma Aldrich). The images were captured and analysed using LSM-880 confocal microscope with Plan-Apochromat 63x/1.40 Oil DIC M27 objective and Fiji software respectively.

**Western Blot**

Western blot analysis was performed using standard protocol. Briefly, proteins were resolved using NuPAGE 4-12% Bis-Tris Protein Gels (ThermoFisher Scientific) and were transferred to the nitrocellulose membrane. Membranes were then blocked for 1 hr with 5% nonfat milk in TBST buffer (50 mM Tris-HCl, 150 mM NaCl and 0.05% Tween 20, pH 7.6) and incubated with primary antibody overnight at 4°C. After incubation, membranes were washed and incubated with HRP-conjugated secondary antibody for 1 hr at room temperature. After washing with TBST, membranes were incubated with ECL substrate (ThermoFisher Scientific) for imaging by gel documentation system (G: Box Chemi XRQ, Syngene, Cambridge, United Kingdom). Rabbit anti-Chk1 Antibody (Cat#A300-298A), Rabbit anti-Phospho RPA32 (S4/S8) Antibody (Cat#A300-245A-M) and Rabbit anti-RPA32 Antibody (Cat#A300-244A-M) were purchased from Universal Biologicals (Passhouse Farm House, Cambridge CB23 3QU). Phospho-Chk1 (Ser345) (133D3) Rabbit mAb (Cat#2348T), Phospho-Chk2 (Thr68) (Cat#2661T), Chk2 Antibody (Cat#2662T) were purchased from Cell signalling Technology (Danvers, Massachusetts, United States). Anti-phospho-ATM (Ser1981) clone 10H11.E12 (mouse monoclonal) (Cat#05-740) was purchased from Merck.

**Treatment with inhibitors**

K562 (2000 cells/well) and CD34^+^ cells (10000 cells/well) were seeded in a clear flat bottom, black polystyrene 96 Well Microplate (Corning Incorporated). Cells were treated with varying concentrations of inhibitors (Sudemycin D6, VE-821, UCN-01 and KU-55933) for 5 days and cell viability was analysed by using resazurin cell viability assay at 560 nm excitation / 590 nm emission filter using SpectraMax i3x (Molecular Devices, San Jose, California, United States). Sudemycin D6 was a kind gift from Professor Thomas Webb, SRI International, California, USA. VE-821 (Cat#SML1415), UCN-01 (Cat#: U6508) and KU-55933 (Cat#SML1109) were purchased from Merck. Percentages of viable cells were calculated by normalizing to the DMSO controls. Synergy score was generated using SynergyFinder (https://synergyfinder.fimm.fi) [[6](#_ENREF_6)] using the Zero Interaction Potency model.

**Statistics**

Statistical analysis was performed using GraphPad Prism 6.01 (San Diego, California, USA). A P-value <0.05 was considered significant. The type of statistical test used and the results including P values, means and standard error of the mean are shown in the figures and figure legends.

**References**

1. Chen L, Chen JY, Zhang X, Gu Y, Xiao R, Shao C, et al. R-ChIP Using Inactive RNase H Reveals Dynamic Coupling of R-loops with Transcriptional Pausing at Gene Promoters. Molecular cell. 2017;68:745-57 e5.

2. Chen L, Chen JY, Huang YJ, Gu Y, Qiu J, Qian H, et al. The Augmented R-Loop Is a Unifying Mechanism for Myelodysplastic Syndromes Induced by High-Risk Splicing Factor Mutations. Molecular cell. 2018;69:412-25 e6.

3. Pellagatti A, Roy S, Di Genua C, Burns A, McGraw K, Valletta S, et al. Targeted resequencing analysis of 31 genes commonly mutated in myeloid disorders in serial samples from myelodysplastic syndrome patients showing disease progression. Leukemia. 2016;30:247-50.

4. Bhatia V, Valdes-Sanchez L, Rodriguez-Martinez D, Bhattacharya SS. Formation of 53BP1 foci and ATM activation under oxidative stress is facilitated by RNA:DNA hybrids and loss of ATM-53BP1 expression promotes photoreceptor cell survival in mice. F1000Research. 2018;7:1233.

5. Bhatia V, Barroso SI, Garcia-Rubio ML, Tumini E, Herrera-Moyano E, Aguilera A. BRCA2 prevents R-loop accumulation and associates with TREX-2 mRNA export factor PCID2. Nature. 2014;511:362-5.

6. Ianevski A, He L, Aittokallio T, Tang J. SynergyFinder: a web application for analyzing drug combination dose-response matrix data. Bioinformatics. 2017;33:2413-5.
