# Supplementary material for: SF3B1 mutations induce R-loop accumulation and DNA damage in MDS and leukemia cells with therapeutic implications
Source: Leukemia. 2020 Feb 19;34(9):2525–30. doi: 10.1038/s41375-020-0753-9 (PMC7449882; doi:10.1038/s41375-020-0753-9)
Supplement: Supplementary file 2 — Table S1 [file 41375_2020_753_MOESM2_ESM.docx]

Table S1: List of patient samples used in this study.

| **Patient ID** | **Disease Subtype** | **SF3B1 Mutation** | **Other Known Mutations** | **Figure number** |
| --- | --- | --- | --- | --- |
| 1 | MDS-RS-MLD | K700E | TET2 G1172Vfs*3; DNMT3A G707Afs*72 | Fig 1C, 1F, S1B, 2B-C, 2E, 2H-I |
| 2 | MDS/MPN | K666N | DNMT3A R882H | Fig 2B-C, 2E, 2H-I |
| 3 | MDS-RS-MLD | K700E | None | Fig 1C, 1F, S3B, 2B-C, 2E |
| 4 | MDS/MPN | K700E | DNMT3A R882H | Fig S2 |
| 5 | MDS-MLD | K700E | None | Fig 1C, 1F |
| 6 | MDS-RS-MLD | K700E | None | Fig 2B-C, 2E, 2H-I |
| 7 | MDS-MLD | None | None | Fig 1C, 1F, S3B |
| 8 | MDS-MLD | None | None | Fig 1C, 1F, S1B |
| 9 | MDS-MLD | None | ETV6 L201P | Fig 1C, 1F |
| 10 | MDS-MLD | None | None | Fig 2B-C, 2E, 2H-I |
| 11 | MDS-MLD | None | None | Fig 2B-C, 2E, 2H-I |
| 12 | MDS-MLD | None | RAD21 A20V | Fig 2B-C, 2E |
