# Supplementary material for: SF3B1 mutations induce R-loop accumulation and DNA damage in MDS and leukemia cells with therapeutic implications
Source: Leukemia. 2020 Feb 19;34(9):2525–30. doi: 10.1038/s41375-020-0753-9 (PMC7449882; doi:10.1038/s41375-020-0753-9)
Supplement: Supplementary file 3 — Supplementary Figure Legends [file 41375_2020_753_MOESM3_ESM.docx]

**Supplementary figure legends**

Fig. S1: Representative images of S9.6 foci detected by immunocytochemistry in (A) SF3B1^K700E^ and isogenic control SF3B1^K700K^ K562 cells and (B) CD34^+^ cells from MDS SF3B1^Mut^, from MDS SF^WT^ and from healthy controls. Green-S9.6; blue- DAPI. Scale bar- 10 µm.

Fig. S2: Characterization of iPSC lines generated from bone marrow CD34^+^ cells of an MDS patient (one iPSC clone harboring an *SF3B1* mutation and one iPSC clone without the *SF3B1* mutation) and from bone marrow CD34^+^ cells of a healthy control. (A) Confirmation of *SF3B1* mutation by Sanger sequencing. (B) Confirmation of clearance of Sendai virus reprogramming factors using real-time quantitative PCR analysis for each transgene-containing virus. Healthy control CD34^+^ cells infected with Cytotune sendai virus two days earlier were used as positive control. iPSC lines show the presence of B2M control gene expression, and no expression of the reprogramming virus genes. (C and D) Analysis of pluripotency markers (SSEA4, TRA 1-81 and TRA 1-60) by (C) immunocytochemistry and (D) flow cytometry. (E) Differentiation of iPSC clones into the three germ layers (mesoderm, endoderm and ectoderm) *in vitro*.

Fig. S3: Representative images of γ-H2AX foci as detected by immunocytochemistry in (A) SF3B1^K700E^ and isogenic control SF3B1^K700K^ K562 cells (B) CD34^+^ cells from MDS SF3B1^Mut^, from MDS SF^WT^ and from healthy controls. Red- γ-H2AX; blue- DAPI. Scale bar- 10 μm.

Fig. S4: Western blot analysis of γ-H2AX levels in SF3B1^K700K-EV^, SF3B1^K700E-EV^, and SF3B1^K700E-RNaseH-WT^ K562 cells (n=3).

Fig. S5: Effect of SF3B1 mutation on ATR and ATM signaling pathways. (A) Western blot analysis of phospho-Chk1(S345) levels in SF3B1^K700K-EV^, SF3B1^K700E-EV^ and SF3B1^K700E-RNaseH-WT^ K562 cells (n=2). (B) Western blot analysis of phospho-ATM(S1981), phospho-Chk2(T68) and phospho-RPA32(S4/8) levels in SF3B1^K700K^ and SF3B1^K700E^ K562 cells (n=3).

Fig. S6: (A) Viability of SF3B1^K700K^ and SF3B1^K700E^ K562 cells treated with VE-821 (n=3). (B) Viability of SF3B1^K700K-EV^, SF3B1^K700E-EV^ and SF3B1^K700E-RNaseH-WT^ K562 cells treated with varying concentrations of UCN-01. IC_50_ obtained for SF3B1^K700K-EV^, SF3B1^K700E-EV^ and SF3B1^K700E-RNaseH-WT^ K562 cells are 132.3±1.06 nM, 64.29±1.08 nM and 96.92±1.11 nM respectively (n=3) (C) Viability of SF3B1^K700K^ and SF3B1^K700E^ K562 cells treated with increasing concentrations of KU-55933. IC_50_ obtained for SF3B1^K700K^ and SF3B1^K700E^ K562 cells are 16.5±1.05 and 19.35±1.12 nM respectively (n=3).

Fig. S7: (A and B) Visualization of 2D synergy maps showing overall synergy scores (δ-score) for combination treatment of sudemycin D6 and UCN-01 on (A) SF3B1^K700K^ and (B) SF3B1^K700E^ K562 cells. (C and D) Visualization of 2D synergy maps showing overall synergy scores for combination treatment of sudemycin D6 and VE-821 on (C) SF3B1^K700K^ and (D) SF3B1^K700E^ K562 cells (n=2). A positive synergy score (δ-score) (Red) denotes synergy.
