# Supplementary figures and images for: SF3B1 mutations induce R-loop accumulation and DNA damage in MDS and leukemia cells with therapeutic implications
Source: Leukemia. 2020 Feb 19;34(9):2525–30. doi: 10.1038/s41375-020-0753-9 (PMC7449882; doi:10.1038/s41375-020-0753-9)

## Slide 1
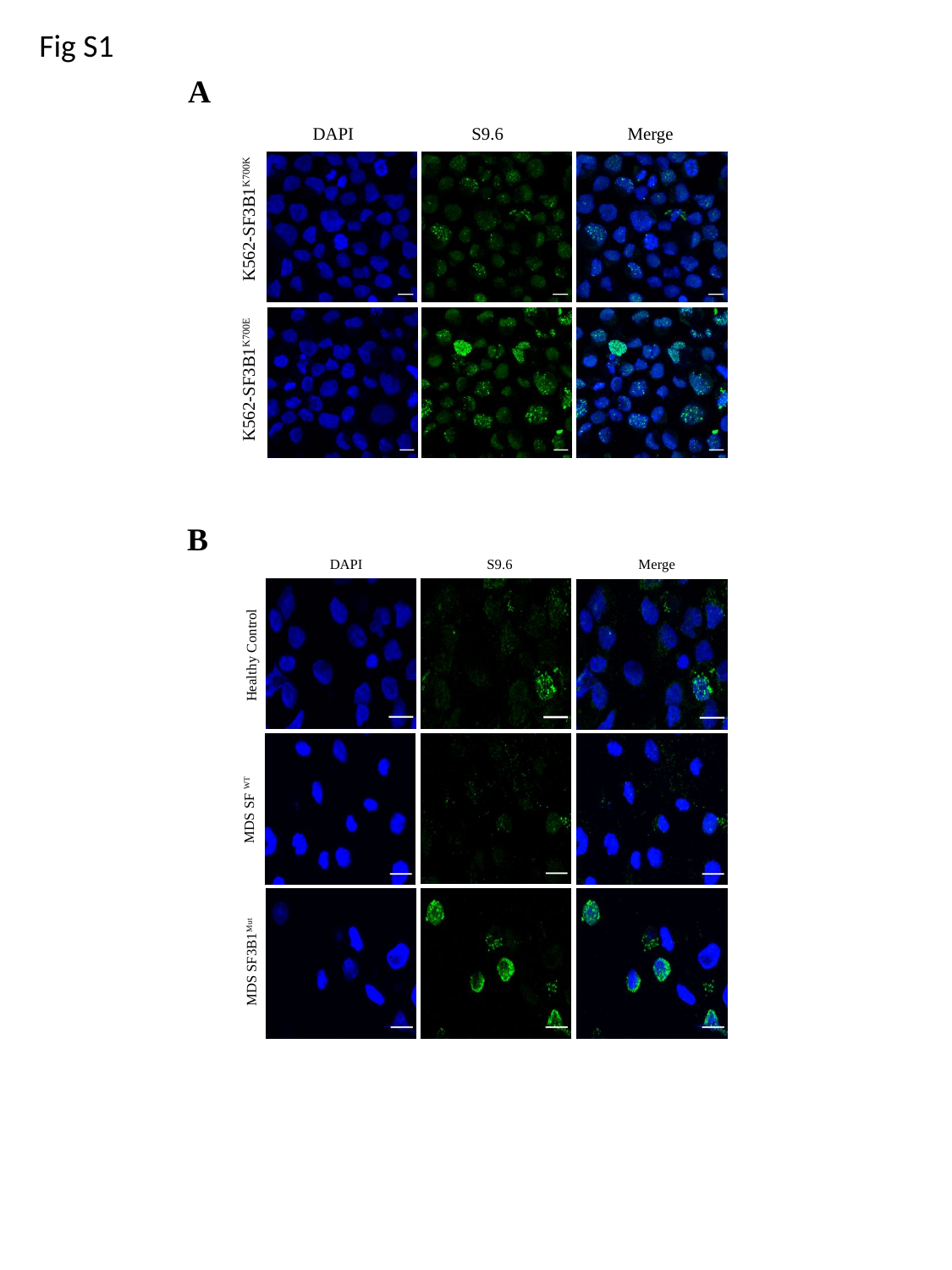

Fig S1
A
Merge
S9.6
DAPI
K562-SF3B1K700K
K562-SF3B1K700E
B
DAPI
S9.6
Merge
Healthy Control
MDS SF WT
MDS SF3B1Mut

Supplement: Supplementary file 4 — Figure S1 [file 41375_2020_753_MOESM4_ESM.pptx]

## Slide 1
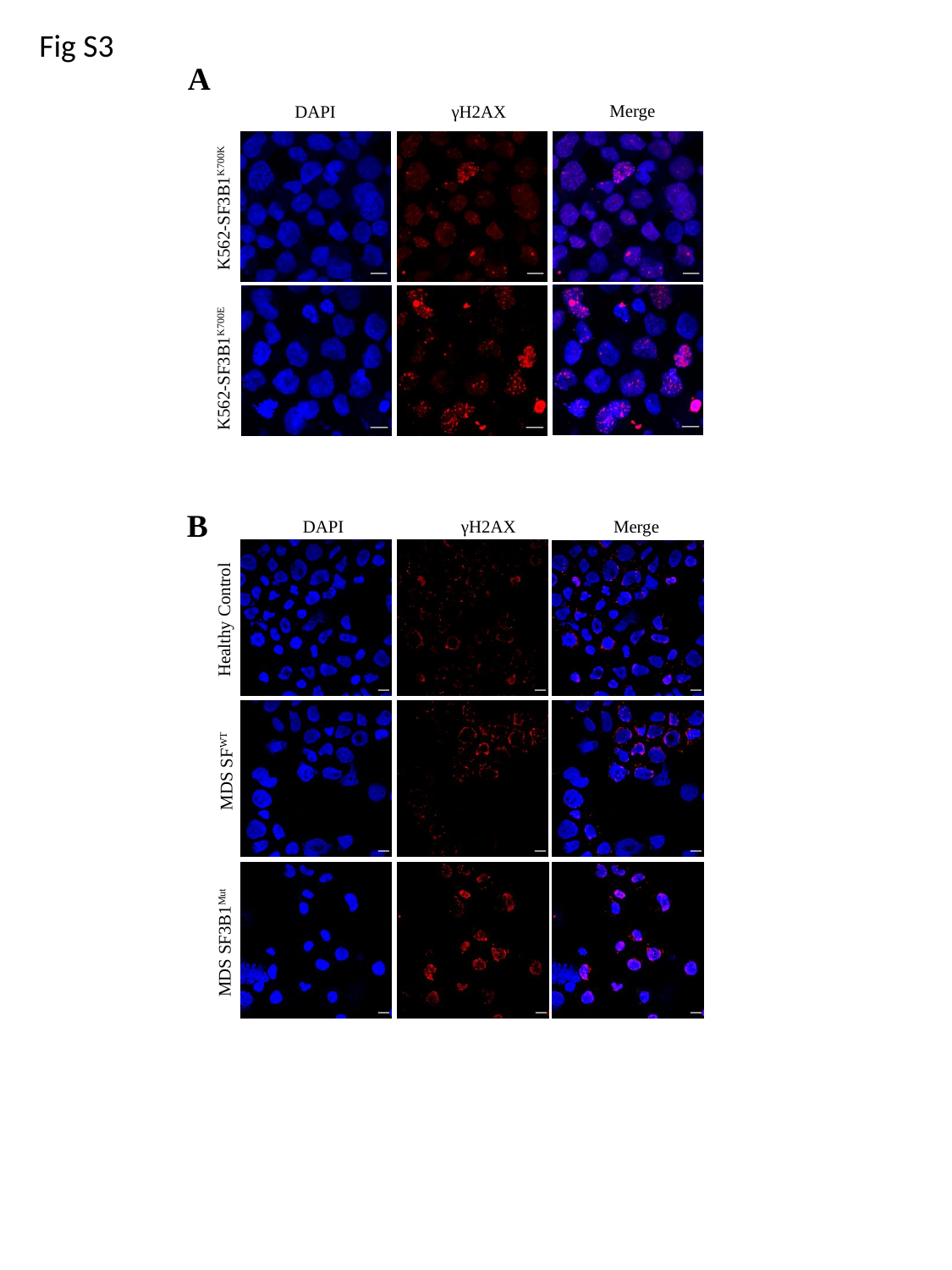

Fig S3
A
Merge
γH2AX
DAPI
K562-SF3B1K700K
K562-SF3B1K700E
B
DAPI
γH2AX
Merge
Healthy Control
MDS SFWT
MDS SF3B1Mut

Supplement: Supplementary file 6 — Figure S3 [file 41375_2020_753_MOESM6_ESM.pptx]

## Slide 1
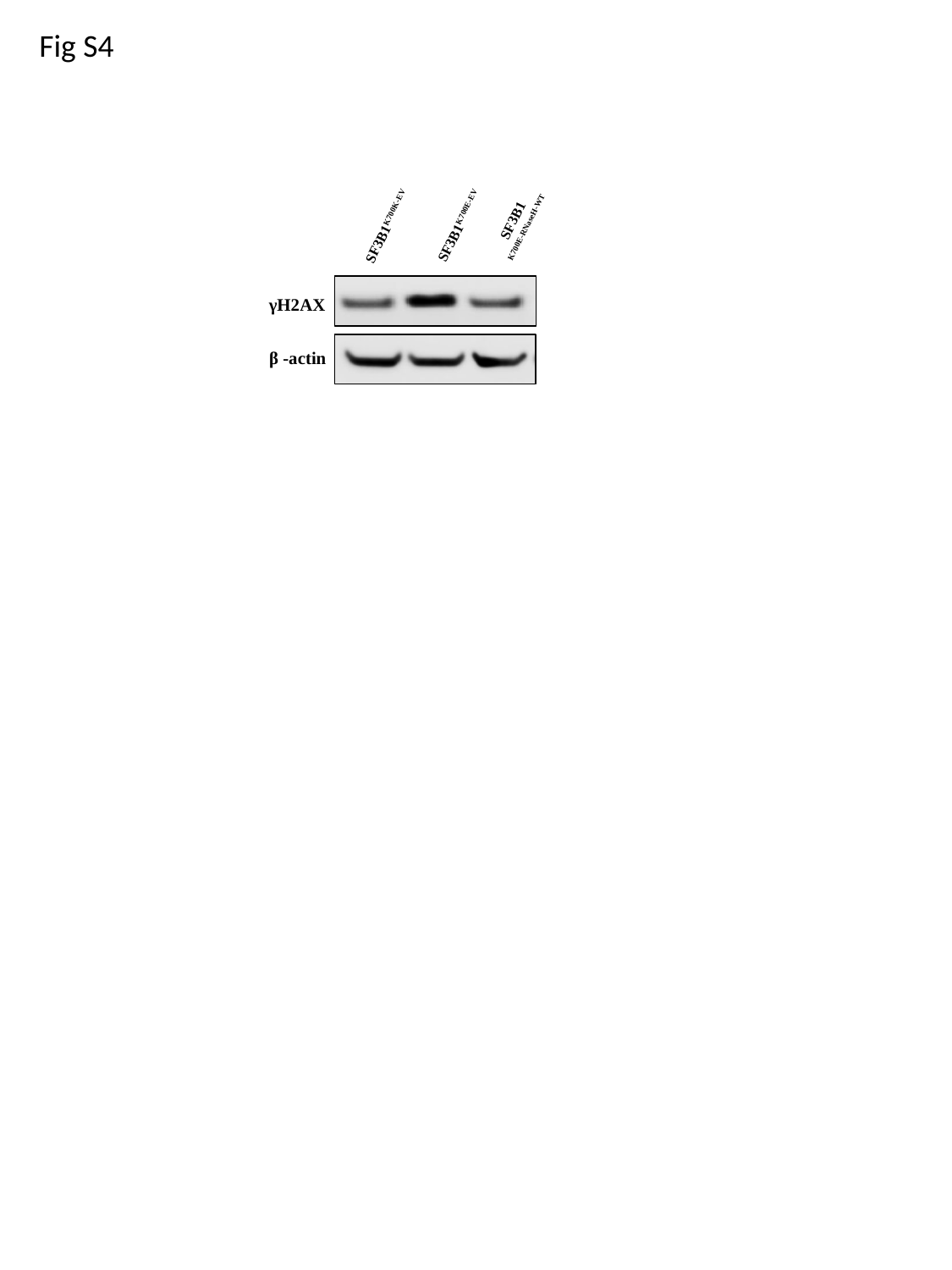

Fig S4
SF3B1K700K-EV
γH2AX
β -actin
SF3B1K700E-EV
SF3B1
K700E-RNaseH-WT

Supplement: Supplementary file 7 — Figure S4 [file 41375_2020_753_MOESM7_ESM.pptx]
