# Supplementary material for: SF3B1 mutations induce R-loop accumulation and DNA damage in MDS and leukemia cells with therapeutic implications
Source: Leukemia. 2020 Feb 19;34(9):2525–30. doi: 10.1038/s41375-020-0753-9 (PMC7449882; doi:10.1038/s41375-020-0753-9)
Supplement: Supplementary file 5 — Figure S2 [file 41375_2020_753_MOESM5_ESM.pptx]

## Slide 1
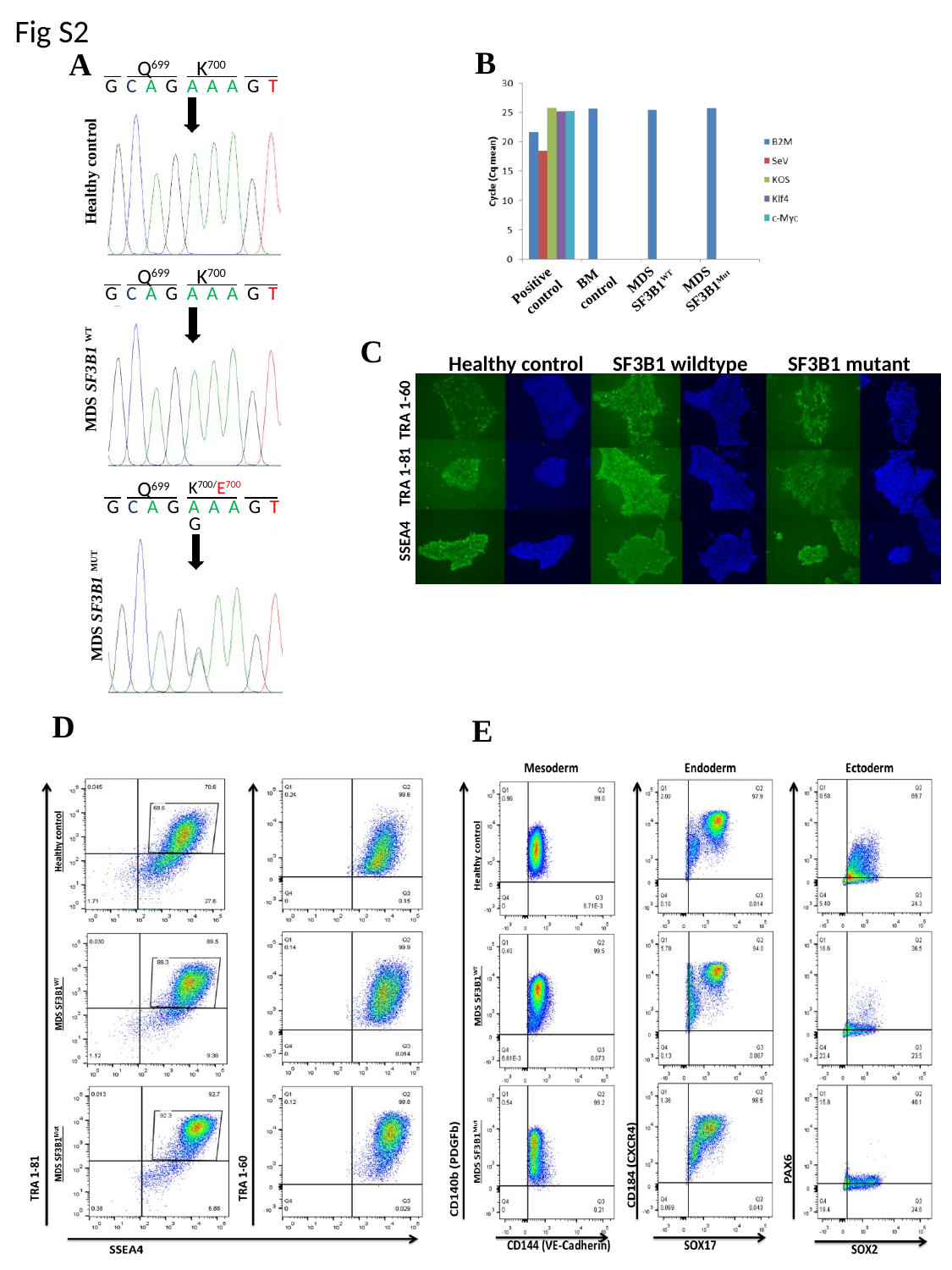

Fig S2
B
MDS
SF3B1WT
BM
control
MDS
 SF3B1Mut
Positive
control
A
Q699
K700
G C A G A A A G T
Healthy control
Q699
K700
G C A G A A A G T
MDS SF3B1 WT
Q699
K700/E700
G C A G A A A G T
MDS SF3B1 MUT
G
C
Healthy control
SF3B1 wildtype
SF3B1 mutant
TRA 1-60
TRA 1-81
SSEA4
D
E
