# Supplementary material for: SF3B1 mutations induce R-loop accumulation and DNA damage in MDS and leukemia cells with therapeutic implications
Source: Leukemia. 2020 Feb 19;34(9):2525–30. doi: 10.1038/s41375-020-0753-9 (PMC7449882; doi:10.1038/s41375-020-0753-9)
Supplement: Supplementary file 8 — Figure S5 [file 41375_2020_753_MOESM8_ESM.pptx]

## Slide 1
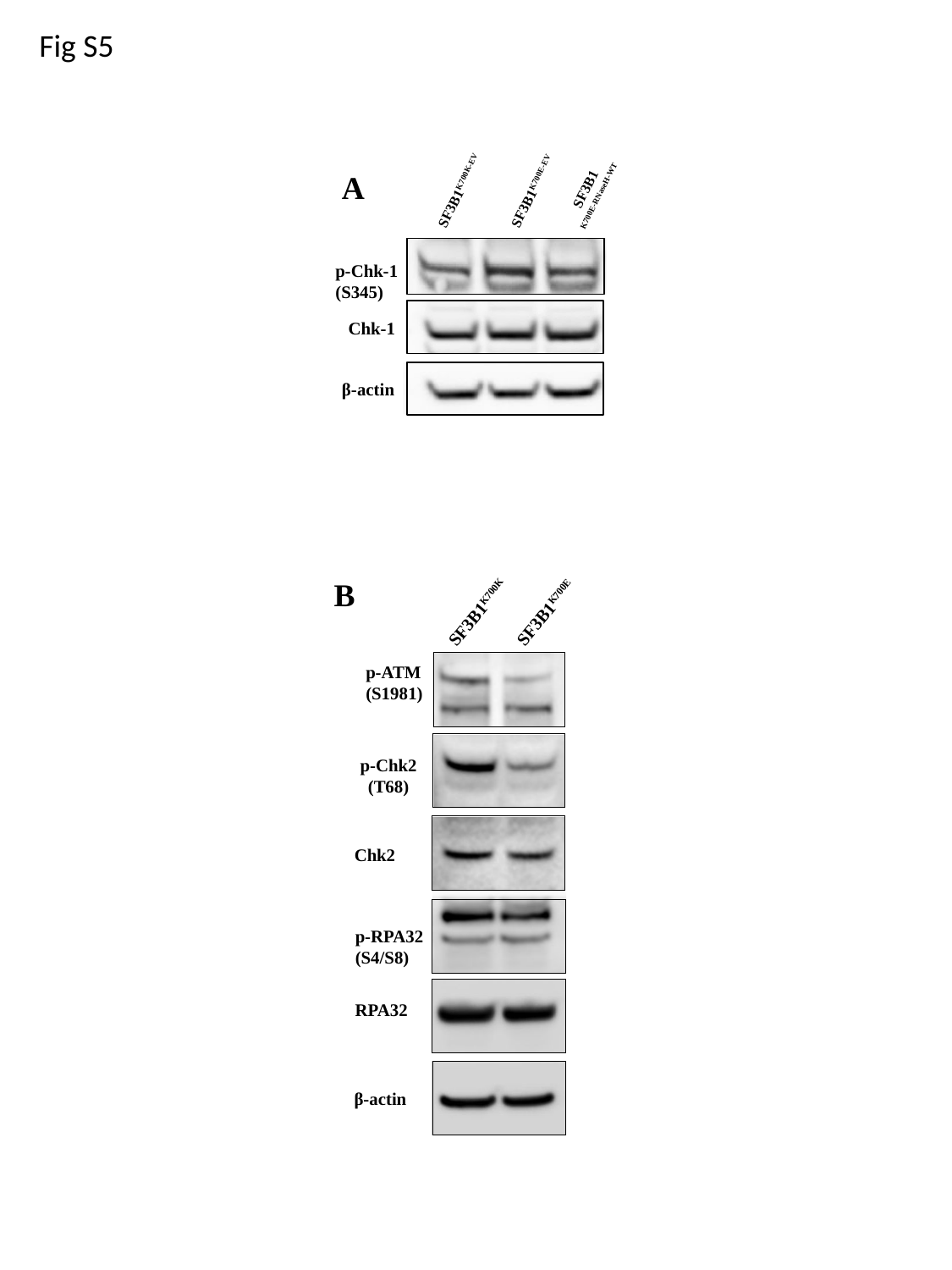

Fig S5
SF3B1K700E-EV
SF3B1K700K-EV
SF3B1
K700E-RNaseH-WT
p-Chk-1
(S345)
Chk-1
β-actin
A
SF3B1K700E
SF3B1K700K
p-ATM
(S1981)
p-Chk2
(T68)
Chk2
p-RPA32
(S4/S8)
RPA32
β-actin
B
