# Supplementary material for: SF3B1 mutations induce R-loop accumulation and DNA damage in MDS and leukemia cells with therapeutic implications
Source: Leukemia. 2020 Feb 19;34(9):2525–30. doi: 10.1038/s41375-020-0753-9 (PMC7449882; doi:10.1038/s41375-020-0753-9)
Supplement: Supplementary file 9 — Figure S6 [file 41375_2020_753_MOESM9_ESM.pptx]

## Slide 1
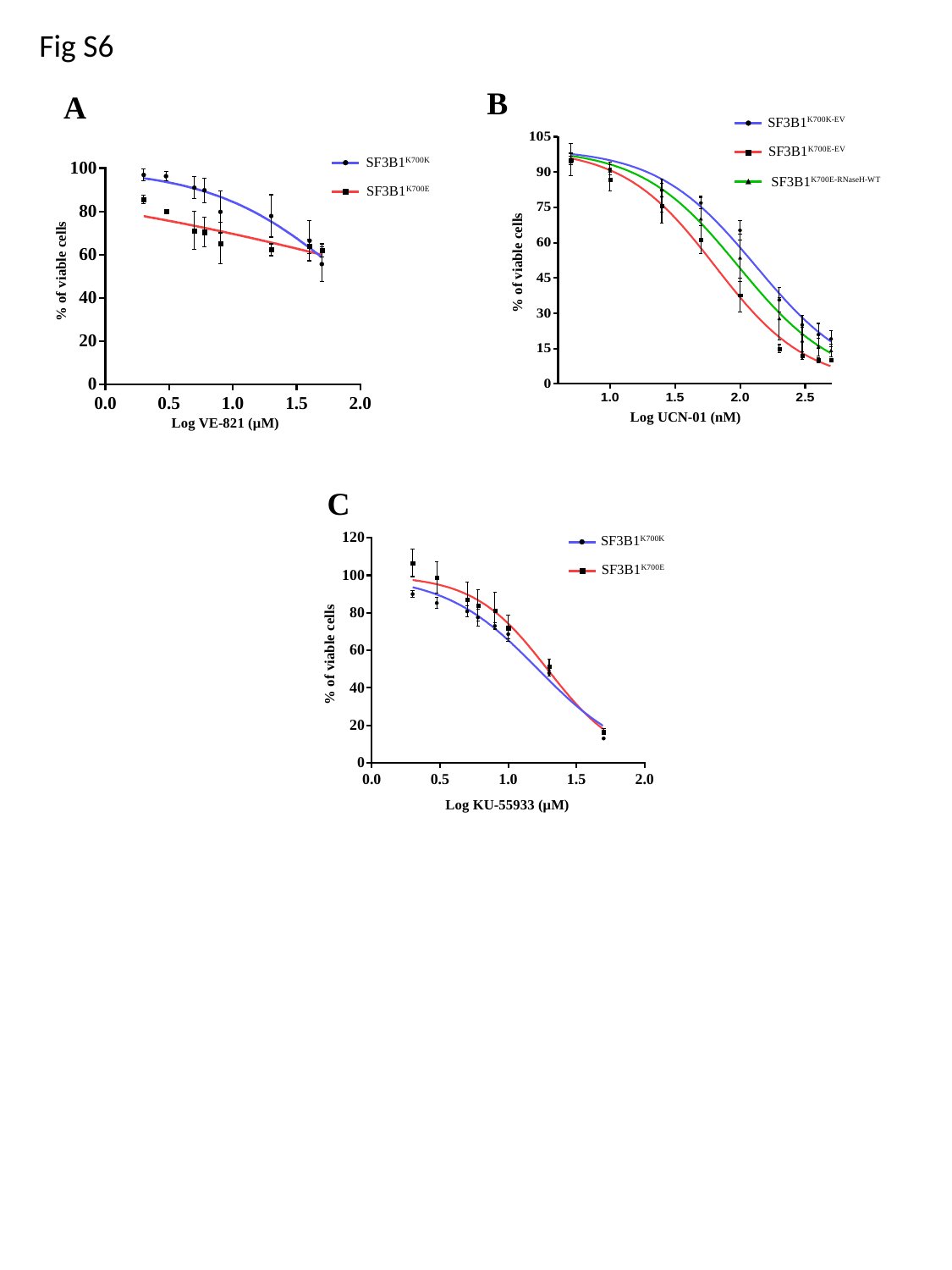

Fig S6
B
A
SF3B1K700K-EV
% of viable cells
Log UCN-01 (nM)
SF3B1K700E-EV
SF3B1K700E-RNaseH-WT
SF3B1K700K
SF3B1K700E
% of viable cells
Log VE-821 (μM)
C
SF3B1K700K
SF3B1K700E
% of viable cells
Log KU-55933 (μM)
