# Supplementary material for: SF3B1 mutations induce R-loop accumulation and DNA damage in MDS and leukemia cells with therapeutic implications
Source: Leukemia. 2020 Feb 19;34(9):2525–30. doi: 10.1038/s41375-020-0753-9 (PMC7449882; doi:10.1038/s41375-020-0753-9)
Supplement: Supplementary file 10 — Figure S7 [file 41375_2020_753_MOESM10_ESM.pptx]

## Slide 1
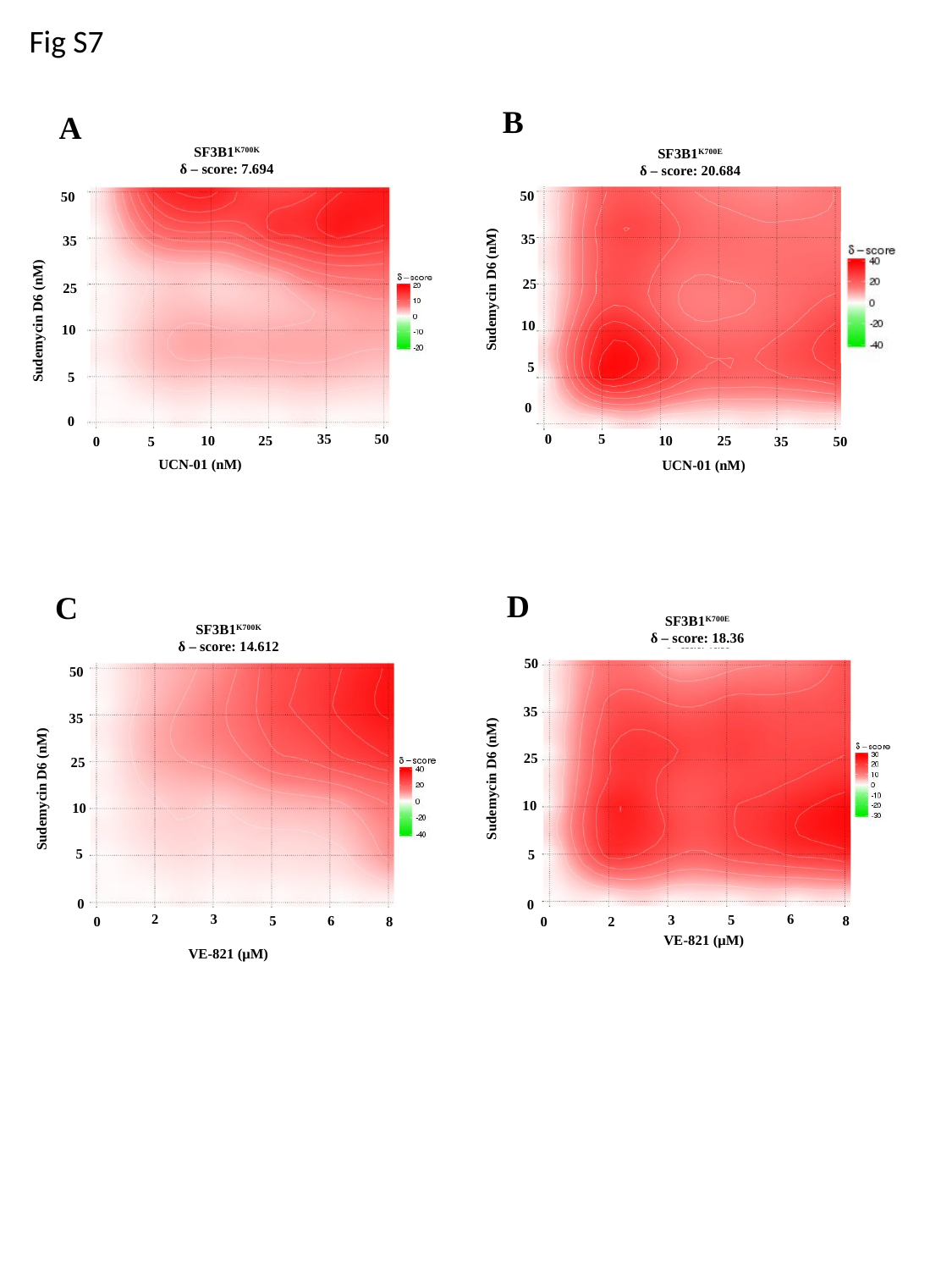

Fig S7
B
A
SF3B1K700K
δ – score: 7.694
50
35
25
Sudemycin D6 (nM)
10
5
0
35
50
25
10
0
5
UCN-01 (nM)
SF3B1K700E
δ – score: 20.684
50
35
25
10
5
0
Sudemycin D6 (nM)
0
5
25
10
50
35
UCN-01 (nM)
D
C
SF3B1K700E
δ – score: 18.36
50
35
25
10
5
0
6
3
5
8
0
2
VE-821 (μM)
Sudemycin D6 (nM)
SF3B1K700K
δ – score: 14.612
50
35
25
10
5
0
2
3
5
6
0
8
Sudemycin D6 (nM)
VE-821 (μM)
